# Influences of hospital information systems, indicator data collection and computation on reported Dutch hospital performance indicator scores

**DOI:** 10.1186/1472-6963-13-212

**Published:** 2013-06-12

**Authors:** Helen A Anema, Job Kievit, Claudia Fischer, Ewout W Steyerberg, Niek S Klazinga

**Affiliations:** 1 Department of Public Health, Academic Medical Center- University of Amsterdam, Room: J2-211, P.O. Box 22660, Amsterdam NL-1100 DD, The Netherlands; 2 Leiden University Medical Center, Leiden, The Netherlands; 3 Erasmus Medical Center, Rotterdam, The Netherlands

**Keywords:** Performance indicators, Health care quality, Reliability, Hospital information system

## Abstract

**Background:**

For health care performance indicators (PIs) to be reliable, data underlying the PIs are required to be complete, accurate, consistent and reproducible. Given the lack of regulation of the data-systems used in the Netherlands, and the self-report based indicator scores, one would expect heterogeneity with respect to the data collection and the ways indicators are computed. This might affect the reliability and plausibility of the nationally reported scores.

**Methods:**

We aimed to investigate the extent to which local hospital data collection and indicator computation strategies differ and how this affects the plausibility of self-reported indicator scores, using survey results of 42 hospitals and data of the Dutch national quality database.

**Results:**

The data collection and indicator computation strategies of the hospitals were substantially heterogenic. Moreover, the Hip and Knee replacement PI scores can be regarded as largely implausible, which was, to a great extent, related to a limited (computerized) data registry. In contrast, Breast Cancer PI scores were more plausible, despite the incomplete data registry and limited data access. This might be explained by the role of the regional cancer centers that collect most of the indicator data for the national cancer registry, in a standardized manner. Hospitals can use cancer registry indicator scores to report to the government, instead of their own locally collected indicator scores.

**Conclusions:**

Indicator developers, users and the scientific field need to focus more on the underlying (heterogenic) ways of data collection and conditional data infrastructures. Countries that have a liberal software market and are aiming to implement a self-report based performance indicator system to obtain health care transparency, should secure the accuracy and precision of the heath care data from which the PIs are calculated. Moreover, ongoing research and development of PIs and profound insight in the clinical practice of data registration is warranted.

## Background

Monitoring the quality of health care by means of performance indicator scores is part and parcel of national health care systems. Performance indicators (PIs) are used to monitor and improve quality and patient safety and to stimulate accountability and market processes in countries worldwide (e.g. USA (http://www.ahrq.com), UK (http://www.hqip.org.uk), and Denmark (http://www.ikas.dk). To play this role effectively, performance indicators need to be reliable and valid measures of health care quality [1-3] particularly when hospitals’ performances are ranked and published in the lay press [4] and/or used to link reimbursement to indicator results [4,5].

National hospital performance indicator programs commonly use PIs that are selected on basis of expert judgment (e.g. medical doctors, patient organizations) and existing scientific evidence [6] about valid relations between health care processes and outcome indicator (e.g. [7]). These PIs have often been successfully implemented in other countries. Due to differences in national healthcare and local hospital organization, copying a performance indicator into another health system does not automatically imply a valid reflection of the underlying health care process that it is intended to measure. Although small pilot studies to assess data collection, indicator computation, data sending and data analyses are commonly executed prior to national PI implementation [6], thorough evaluations of reliability and validity of the PIs after implementation, are scarce [8,9]. Therefore it remains unclear whether such selected PIs can be validly used in the national health care system.

For a PI to be valid, it needs to be composed with the least possible measurement error. Most indicators consist of a numerator (e.g. number of patients that timely received antibiotics prophylaxis) and a denominator specifying the population at risk [10,11] (e.g. the number of patients that should receive the prophylaxis). To compose the numerator and denominator, patients that fit the inclusion criteria need to be identified in the data systems. This selection process is explained in instruction manuals that describe the specific steps that need to be taken. Each step yields a data element, for instance the date of surgery, or a secondary diagnosis (comorbidity). For some steps it is required to select several data elements and as such consist of a set of rules that combine several data elements, the latter increasing the complexity of the process [12].

In many quality indicator programs, (e.g. in the USA: AHRQ, Kaiser Permanente, Veteran Affairs Quality program; Denmark: IKAS; Germany: BQS) the coordinating organizations are responsible for PI data collection and computation, as opposed to programs that rely on self-report of the participating hospitals. They abstract the indicator data from digital administrative (hospital information system) or financial databases using computerized data abstraction algorithms. This approach however, is effective only when the data-systems are identical for all participating hospitals. When hospitals do not have an integral electronic patient record, patient information is stored in several information systems. Moreover, when a country has a liberal software market (US, The Netherlands) and the PIs are based on self-report, coupling of these independent information systems in an attempt to automatically collect the data might be difficult and prone to error due to the various software environments. Although manually selecting the data from all the systems and paper records seems less error-prone, it is very time consuming. It could be assumed, therefore, that hospitals obtain their own, unique, strategy to compute the PI score, which makes comparison of the PI scores difficult. Therefore, we question whether the construction of Dutch hospital information systems that are used to compute PI scores and the extent to which data elements are available and accessible affect the accuracy and precision of the PI scores in a negative way.

To investigate this, we study the Dutch local hospital data-infrastructure, here defined as the availability and accessibility of PI data elements. In 2008, the Dutch government implemented hospital PIs for various disease specific groups (Dutch Health Care Transparency Program; DHTP^a^). All Dutch hospitals are required to report these PIs to the government on a yearly basis, to pursue public disclosure of health care performance, data benchmarking, selective contracting by insurance companies, and decision making processes of patients looking for a healthcare provider. The selection of the indicators was performed by disease specific expert groups of medical doctors, patient organizations, and health care insurers, on the basis of published guidelines and expert opinion. Attempts were made to standardize the structure, process and intermediate outcome indicator definitions, as well as the data collection and indicator computation instructions. These instructions are principally code based, that is, based upon diagnose and procedure codes such as the ICD-9 classification or DRG codes (Diagnosis Related Group codes). Dutch hospitals are independent organizations and free to choose information technology systems for clinical and administrative data. Therefore, developing instructions that are specified for the information system that is used to handle the data is not feasible.

Since Dutch hospitals are solely responsible for reporting the required indicator scores, reliability (precision and accuracy) of the self-reported PIs might be particularly at risk. Thus, given the lack of regulation of the data-infrastructure (information systems that are used) in the Netherlands, and the self-report based indicator scores, one would expect heterogeneity with respect to the data collection and the ways indicators are computed. Moreover, it can be expected that hospitals that have a low level of automation and difficulties to connect all the individual information systems, make use of data that are registered in external databases such as the National Cancer Registry of the Comprehensive Cancer Centers (in Dutch: IKNL; further referred to as CCC).

Together, we aimed to obtain insight in the precision and accuracy of publically available PI scores and the impact that the local hospital data-infrastructure has on these aspect using Dutch indicator scores of two sets of surgical PIs (Table 1 and Additional file 1): Hip and Knee Replacements (further referred to as HR or KR) and Breast Cancer (further referred to as BC). First, to obtain a general idea of the accuracy and precision and second, to investigate the long term pattern of a hospital’s performance, we evaluated the plausibility of the available PI scores of health care delivered between 2008 and 2010. With plausibility we mean the extent to which the indicator score is in line with what we can expect from the health care procedure, on basis of the literature, guideline compliance and audit studies. For example, a process indicator score of 100% that measures the number of patients that timely received surgically related antibiotic prophylaxis might be implausible, as relevant literature on guideline compliance reveals average scores as low as 50% [13-15]. Second, using data obtained through a questionnaire among a sample of Dutch hospitals in 2010, we checked whether the data elements that are required to compute the indicator scores were registered at all. Further, if available, we wanted to know whether these data elements were easy to access or only after time consuming actions, whether hospitals calculated the indicator on basis of the entire population at risk, or whether the PI score was merely estimated. And finally, we investigated the relation between the data-infrastructure (data availability and accessibility), the way hospitals calculated the indicator scores and the plausibility of the submitted indicator score.

**Table 1:** Overview of process and outcome indicators hip and knee replacements and breast cancer

**National performance indicators**				
	Total Hip and Knee replacements *	**S**	**P**	**O**
**2b**	% of patients that was administered thrombosis prophylaxis for 6 weeks to 3 months post-surgery, in case of total hip or knee surgery		X	
**4b**	% of patients that **did not** (2008 & 2009)/ **did** (2010) receive a homologue blood transfusion, in case of total hip or knee surgery		X	
**5b**	% of patients that was administered antibiotics perioperatively		X	
**5c**	% of patients that was administered antibiotics 15 to 60 min. prior to surgery or to blood emptiness		X	
**5d**	% of patients with a deep wound infection after a total hip or knee replacement			X
	Breast Cancer **	**S**	**P**	**O**
**1**	% of patients who were seen by a breast cancer nurse specialist preoperatively		X	
**2**	% of patients that was reviewed preoperatively in a multi-disciplinary team meeting		X	
**3**	% of patients with a non-radical primary tumor resection			X
**4**	% of surgeons in the surgery department that perform surgical treatments of breast tumors	X		
**5**	% of patients that are operated within 4 weeks after the final lab results are known		X	
**6a**	% of patients with local recurrences within 5 years after breast-conserving surgery			X
**6b**	% of patients that have local recurrences within 5 years after ablative breast surgery			X
**7**	% of patients with a breast tumor that was postoperatively reviewed in a documented multi-disciplinary team meeting	X		

* Note: 5 yes/no “Hip/Knee structure indicators” are omitted from the table as they were not included in the current study; ** Indicators 1, 2 and 7 were removed from the indicator set in 2009, 4 in 2011; *S* structure, *P* process, *O* intermediate outcome; The PIs consist of numerators and denominators that each are composed of several variables according to combinatory logic that is described in instruction manuals. See for details of numerators and denominators Additional file 1.

## Methods

### Study design

A cross-sectional mixed methods design, using both qualitative and quantitative data from three different sources was used to explore the effect of the data-infrastructure on the accuracy and the precision of the PI scores.

### Study population

The study population consisted of national hospitals (in 2010) of which 24 were small hospitals (< 320 beds), 48 were intermediate (320–627 beds), and 28 were large hospitals (> 627 beds), 27 were teaching hospitals and seven were university hospitals. A teaching hospital is a large hospital that is approved of training medical doctors, without being affiliated to a university.

In total 42 national hospitals (42%) gave informed consent^b^ to participate in the study and returned the questionnaire. This representative sample included five small (< 320 beds), 25 intermediate (320–627 beds) and nine large hospitals (> 627), 13 were teaching hospitals and two were university hospitals.

### Data sources

#### DHTP performance indicator database (A)

In 2009, 2010 and 2011, approximately 100 Dutch hospitals submitted performance indicator scores (web-based entry tool), indicative of care delivered the year before (2008, 2009 and 2010 respectively) at the DHTP.

#### Additional reliability data DHTP (B)

Besides the indicator scores, the DHTP requires hospitals to upload information (self-report) regarding the reliability of the submitted indicator scores. One dichotomous item specifically targets how the hospital computed the indicator score, i.e. by means of an integral calculation of the total population at risk (further referred to as “calculation”), or by merely estimating it (further referred to as “estimation”). “Estimating” implies either an extrapolation of scores based on a small sample or based on locally implemented protocols. This item was used for our question about the strategy that hospitals used to compose the indicator and was only available for the hospitals that were enrolled in the qualitative part of our study in 2010.

#### Web-based questionnaire (C)

The short web-based questionnaire targeted the hospital’s local data infrastructure (collection of computer programs and databases) and was setup to answer our questions about the data infrastructure (what is the data availability and data accessibility?). Data availability was dichotomously assessed; a hospital confirmed whether the required information was registered somewhere in the hospital or not. Data accessibility was divided into three categories: 1) Automatically (Aut) accessible, 2) partly automatically accessible (Partly Aut), 3) or manually accessible (Man). *Automatically* accessible refers to data elements that are stored within a computer information system, can be easily reviewed (“only a few mouse clicks away”) and can be abstracted by means of computerized search algorithms (Queries). *Partly automatically* accessible refers to data elements that are available in electronic systems, can be reviewed easily, but cannot be abstracted by means of a computerized search algorithm as some manual actions are required. *Manually accessible* refers to data elements that are available but only through labor intense data handlings such as medical chart reviews.

The BC questionnaire differed from that of the hip and knee questionnaire on the accessibility items. In the Netherlands, the accessibility of performance indicators can be dependent upon external organizations. For example, the data of BC care is simultaneously collected by the Dutch CCC. Data-managers of these centers retrospectively visit a hospital and collect and register cancer specific information (tumour type, health status etc.) in the national cancer database (NKR). When uploading the DHTP indicators, hospitals can decide whether to use their own calculated indicator scores or those calculated by the CCC. As part of our interest in indicator composition strategies, we additionally added an item in the BC questionnaire about whether hospitals collected and calculated their own BC indicator scores, or whether indicator scores from the CCCs was used.

### Analyses

In the current study we used the following variables of interest:

1) The plausibility of the PI score: *plausible score* (PS): a score larger than 0% and smaller than 100%; *implausible score* (IS): a score of 0% or 100%.

2) Data-availability scores: *all required data elements available* (A)*; not available* (NA).

3) Data-accessibility scores: *data elements automatically available*, *partly automatically available*, *manual available*; and *easy accessible* (EA)*, difficult to access (*DA).

4) Indicator Computation scores: PI score based on *integral calculation of population* (Cal); *estimation of PI score* (Est); *CCC calculation:* indicator score calculated by the CCC (BC only).

**Figure 1:**
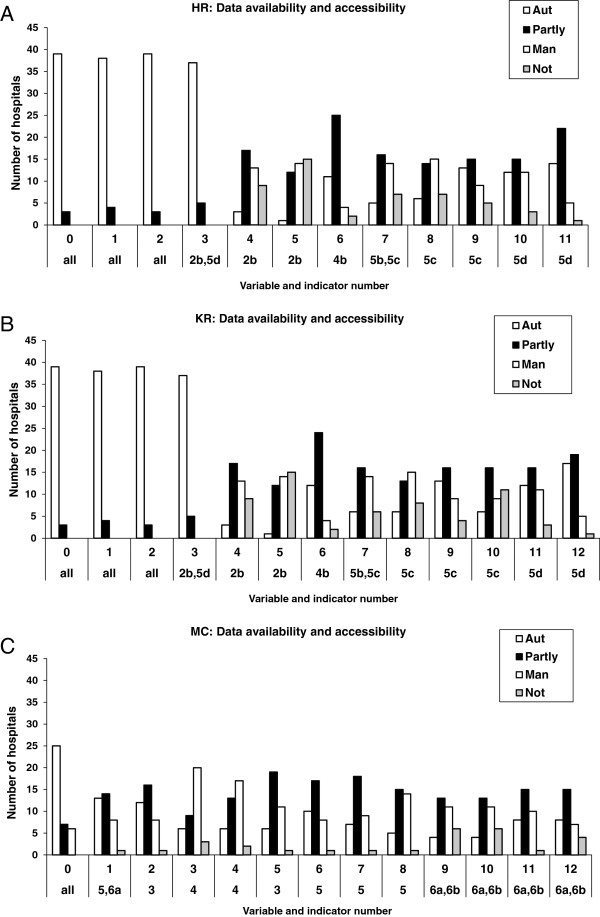
**Reported data infrastructure of the orthopedic and oncology sets" as short and concise descriptive titel of Figure 1ABC.** ABC: Reported data infrastructure of the orthopedic and oncology sets. AUT = fully automatic accessible, Partly = partly automatic, partly manually accessible, Man = manually accessible, NOT = not available; HR = Hip replacement, KR = Knee replacement, BC = Mammacarcinoma; Numbers 1, 2, 3 etc. = numbers that indicate the indicator variable which is part of the indicator set; 2b 4b 5b etc. = the unique number of the indicator.

To get an overall idea of the plausibility of the submitted national PI scores we first provide an overview of the characteristics (means and SDs) of the PI scores that are available in the DHTP database (Data source A) and judge the plausibility (that is, perfect performance of 100% or 0%) and compare the indicator scores with what could be expected on basis of the literature (qualitative approach). Secondly, to answer our question regarding the data infrastructure we analyze the web-questionnaire items (Data Source C) and present frequency results of data-availability and data accessibility scores. Finally, to obtain information about the relation between the data-infrastructure, the procedures that hospitals use to compose the PIs (obtained from Data Source B) and the resulting PI scores, we calculated 2 × 2 and 2 × 3 Chi-square tests. Results are presented separately for the Hip replacements indicator set and the Breast Cancer indicator set. As Hip and Knee replacements yield fairly similar scores (see Table 2 and Figure 1B), we present the data of HR only.

**Table 2:** Results of the descriptive statistics of hip and knee replacements and Mammacare

		**2008**				**2009**				**2010**				
	**PI**	**N**	**M**	**SD**	**Range**	**N**	**M**	**SD**	**Range**	**N**	**M**	**SD**	**Range**	**IS**
**HR**	**2b**	64	99.92	0.602	95 –100	95	99.8	0.949	93 – 100	94	99.9	0.30	98 – 100	53
	**4b**	52	91.27	22.79	0 – 100	91	90.6	15.24	0 - 100	93	16.13	26.58	0 – 100	6
	**5b**	65	100	0.000	100 - 100	96	99.7	1.402	93 - 100	94	101.0	15.65	70 – 100	53
	**5c**	59	97.38	14.00	0 – 100	94	98.0	5.827	66 - 100	93	98.9	16.98	64 – 100	37
	**5d**	60	0.816	0.740	0 – 2.7	93	0.719	0.674	0 – 2.75	93	0.754	0.804	0 – 4	5
**GM**		**60**	**78**	**8**	**/**	**94**	**78**	**5**	**/**	**93**	**63**	**12**	**/**	**37**
**KR**	**2b**	63	99.92	0.663	95 - 100	94	99.8	0.834	93 - 100	93	100	0.246	98 – 100	52
	**4b**	54	91.17	25.72	0 - 100	89	95.6	10.99	0 - 100	92	11.65	27.15	0 – 100	7
	**5b**	64	100	0.00	100 - 100	95	99.8	1.101	92 - 100	93	99.6	2.342	78 – 100	52
	**5c**	59	96.84	15.71	0 - 100	93	97.8	6.52	60 - 100	92	96.8	8.979	49 – 100	39
	**5d**	59	0.50	0.649	0 – 3	92	0.554	0.631	0 – 3.2	92	0.544	0.616	0 – 3.3	6
**GM**		**60**	**78**	**9**	**/**	**93**	**79**	**4**	**/**	**92**	**62**	**8**	**/**	**37**
**BC**	**1**	68	100	5.055	75 – 100	/	/	/	/	/	/	/	/	/
	**2**	68	100	3.200	85 – 100	/	/	/	/	/	/	/	/	/
	**3**	66	9.675	5.464	0 – 24	95	9.215	4.733	0 – 29	94	7.279	4.026	0.95 - 23	0
	**4**	68	41.4	12.68	10 - 75	95	38.5	11.53	10 - 60	/	/	/	/	/
	**5**	63	90.48	14.92	17 - 100	95	89.2	10.30	51 - 100	94	88.9	11.85	34 – 100	0
	**6a**	57	2.130	2.247	0 -11	89	1.748	1.945	0 - 9	93	1.490	1.703	0 – 8	0
	**6b**	57	2.700	2.838	0 - 11	90	2.581	2.522	0 - 11	93	2.455	2.351	0 - 10	0
	**7**	65	100	5.568	74 - 100	/	/	/	/	/	/	/	/	/
**GM**		**64**	**56**	**6**	**/**	**93**	**28**	**6**	**/**	**94**	**25**	**5**	**/**	**0**

Note: *PI* Performance Indicator, *N* number of hospitals, *M* mean, *SD* standard deviation, *GM* grand mean, *IS* Implausible Score (100% or 0% score three consecutive years), *HR* Hip replacements, *KR* Knee replacements, *BC* Breast Cancer; Number of hospitals vary slightly throughout the text due to differences in how hospitals are enrolled in the study (concerns vs separate hospitals). Note 2: No perfect score defined for Performance indicator BC 4.

## Results and discussion

### Hip replacement indicators

#### Data source A: Plausibility of the reported scores

All PIs have high averages (ceiling effect; see Table 2) and PIs 4b (2008, 2009, 2010) and 5c (2008) have large ranges, from 0% to 100%. A 0% compliance to blood transfusion guidelines can be regarded as implausible, being most likely an error. This might be explained by a change in the indicator definition of 4b in 2010; from “no homologue blood transfusions” to “homologue blood transfusions”. Homologue blood transfusions increase the risk of blood borne infections and thus need to be reduced (Dutch Institute for Health Care improvement CBO guideline hip and knee arthrosis 2007 [16]).

As described in the introduction, high averages of the PIs can be regarded as unrealistic, particularly when performance is consistently high for several consecutive years. We determined the number of 100% scores (and 0% scores for outcome indicator 5d; further referred to as “implausible score”) for each year and calculated the number of hospitals that maintained a perfect score for three consecutive years. Implausible scores were rather consistent for the indicators 2b, 5b and 5c (53, 53 and 37 times; see Table 2). Also, five hospitals scored an implausible 0% of post-operative deep wound infections in three consecutive years (outcome indicator 5d).

#### Data source C: Heterogeneity of reported local data infrastructure

The questionnaire revealed that, averaged over all indicators, 26 hospitals (62%) reported to have all the required data elements that are necessary for the calculation of the PI available at the time of study (Figure 1A). When at least one data element was missing (data element unavailable; n = 16), eight hospitals indicated to miss one or two data elements, four hospitals missed three or four data elements and four hospitals missed five or six data elements.

Most of the HR data elements were on average automatically accessible (43%) or partly automatically accessible (30%), whereas 17% was only manually accessible (10% of the information was not available). The data elements that were most frequently indicated to be fully automatic accessible were necessary for computing the denominator (category ALL: elements: 0 patient identification number, 1 financial code hip replacement, 2 procedure code hip replacement, 3 date of hip replacement surgery), whereas the data elements that are less easily accessible are necessary for the numerators of the various indicators.

#### Data source ABC: Relation between computation methods, data collection and PI score

To investigate the relation between the data availability and computation methods we first divided the hospitals in two separate categories; those who indicated to have at least one required data element for a certain indicator unavailable (NA = not available; in total 15 hospitals) and those who had all required data elements for an indicator available (A = available; in total 27 hospitals). Chi square tests revealed that the computation method that hospitals choose is significantly associated with the data availability. That is, when data were unavailable, indicator scores were on average more often estimated as compared to calculated (Est = 91%; Cal = 9%). When data were available this pattern was reversed (Est = 42%; Cal = 58%; χ^2^ (1) = 51.71, p < 0.01. However, nearly half of the indicator scores were estimated even when all the required data elements were available.

A similar association might be found between data accessibility and the composition method. To test this association we divided the data set of our sub sample with all data elements Available (N = 27) in an “Easy Access” (EA) and “Difficult Access” (DA) category for each indicator separately. We assigned 1, 2 or 3 points to the accessibility scores Automatic, Partly Automatic and Manual and averaged the scores per indicator. An EA score for a certain indicator was obtained when the average accessibility score was below 1.5, or else it was given a DA score.

Table 3 revealed that the 27 hospitals reported to have all data available of in total 186 indicators (76 + 73 + 32 + 5). Most indicators were classified as “Difficult to access” (76 + 73 = 149) as compared with the “Easy access” category (32 + 5 = 37). Again Chi square tests revealed a significant relation between composition strategy and data accessibility as reported for the data availability χ^2^ (1) = 42.35, p < 0.01), but this relation was different as compared to the NA category. That is, when data was difficult to access an almost equal number of indicators were based upon integral calculations and estimations (Cal = 51%; Est = 49%). When data was easy to access, the percentage of scores that were based upon an estimation decreased considerably (Cal = 86%; Est = 5%).

**Table 3:** Number of Hip Replacement indicators with plausible, implausible and missing values for the separate indicator computation and data collection strategies

				**2b**	**4b**	**5b**	**5c**	**5d**	**Total**
**NA**		**CAL**		**2**	**0**	**0**	**0**	**1**	**3**
		**EST**		**12**	**2**	**7**	**9**	**0**	**30**
**A**	**DA**	**CAL**	**Total**	**12**	**22**	**12**	**10**	**20**	**76**
			**IS**	11	4	11	7	7	40
			**PS**	1	18	1	3	12	35
			**MV**	0	0	0	0	0	0
		**EST**	**Total**	**24**	**5**	**21**	**22**	**1**	**73**
			**IS**	23	3	20	21	1	68
			**PS**	1	2	1	1	0	5
			**MV**	0	0	0	0	0	0
	**EA**	**CAL**	**Total**	**1**	**11**	**4**	**3**	**13**	**32**
			**IS**	1	1	4	2	4	12
			**PS**	0	10	1	1	11	23
			**MV**	0	0	0	0	0	0
		**EST**	**Total**	**1**	**0**	**1**	**2**	**1**	**5**
			**IS**	1	0	0	1	0	2
			**PS**	0	0	0	1	1	2
			**MV**	0	0	0	0	0	0

Note: *IS* Implausible (100% or 0%) Score, *PS* Plausible score (<100%), *MV* missing value, *NA* Not available, *EA* easy access category, *DA* Difficult Access category, *Cal* calculation, *Est* Estimation; All data in frequencies. Scores are frequencies at the level of indicators, and not at hospital level, to avoid excluding hospitals with missing values from the analyses.

Next, we tested the association between the data infrastructure of available data and the plausibility of the indicator scores. Overall more implausible (IV) than plausible scores (PV) were observed (A: IS = 65%, PS = 35%). However, easy accessible data yielded more plausible indicator scores (PS = 64%; IS = 38%) as compared to the difficult access indicators (PS = 27%; DA, IS = 73%; χ^2^ (1) = 24.64, p < 0.01).

Finally, to see whether the plausibility of the indicator scores was more associated to the composition strategy as compared to the data infrastructure we summated across all data-infrastructure categories (NA + DA + EA) all the indicators that were calculated and checked whether they were implausible or plausible scores and did the same for the estimated indicators. The Chi square test revealed a strong significant association (χ^2^ (1) = 59.05, p < 0.01). Implausible indicator scores were more often estimated (91%) as compared to calculated (38%) whereas plausible scores (scores < 100%) were more often calculated (62%) as compared to estimated (9%).

### Breast cancer indicators

#### Data source A: Plausibility of the reported scores

In contrast to the orthopedic data, implausible perfect scores in three consecutive years could not be identified.

#### Data source C: Heterogeneity of reported local data infrastructure

The questionnaire revealed that, averaged over all indicators, one third of the hospitals (13 out of 41) have all the required data elements available (Figure 1C). When having registered some of the required data elements (27 hospitals), eight hospitals indicated to miss one or two data elements, one hospital missed three data elements and another hospital reported to miss ten of the required twelve data elements. Most of the information was on average partly automatically accessible (39%), whereas 24% was automatically and 30% only manually accessible (7% was not available). The data element that was indicated as automatically accessible the most frequent (element 0: patient identification number) was necessary for computing the denominator.

#### Data source ABC: Relation between computation methods, data collection and PI score

In contrast to the results of the orthopedic indicator sets, only one hospital indicated to have estimated the indicator score. Therefore we focused on the hospitals choice to use the CCC to compose the indicator score, or to use own scores. As can be observed in Table 4 (total column) most indicator scores were, on average, based upon CCC data when data was not available (NA; CCC = 18 indicators, OWN = 3 indicators). To test whether the choice to use the CCC data was associated with the accessibility of available data we determined for each indicator and access category separately how often a hospital used the CCC score and tested the association with a 2 × 2 (DA/EA vs. OWN, CCC) chi square test. The chi square test revealed that the accessibility did not seem to influence the choice for the CCC (DA: OWN = 46%, CCC = 54%; EA: OWN = 39%, CCC =61%; χ^2^ (1) = 0.74, p = 0.39). As was described above, implausible perfect scores on the breast cancer indicators are scarce. Therefore we did not further investigate the effect of the data infrastructure and composition strategy on the indicator score.

**Table 4:** Number of breast cancer care indicators for the separate indicator computation and data collection strategies

			**1**	**2**	**3**	**4a**	**4b**	**Total**
**NA**		**Total**	1	3	3	8	8	**23**
		**OWN**	0	3	0	0	0	**3**
		**CCC**	1	0	3	7	7	**18**
		**MV**	0	0	0	1	1	**2**
**A**	**DA**	**Total**	28	32	28	29	28	**145**
		**OWN**	16	28	14	3	4	**65**
		**CCC**	12	4	13	25	23	**77**
		**MV**	0	0	1	1	1	**3**
	**EA**	**Total**	8	4	7	5	5	**29**
		**OWN**	4	2	3	1	1	**11**
		**CCC**	4	1	4	4	4	**17**
		**MV**	0	1	0	0	0	**1**

Note: *OWN* Self-calculated, *CCC* calculated by Collective Cancer Center, *MV* missing value, *NA* Not available, *EA* easy access category, *DA* Difficult Access category, *Cal* calculation, *Est* Estimation; Scores are frequencies at the level of indicators, and not at hospital level, to avoid excluding hospitals with missing values from the analyses.

### Discussion

#### Summary

The current study revealed that the Dutch PI hospital data infrastructure is heterogeneous, and the reported performance data under investigation can be regarded as largely implausible, particularly those of the Hip and Knee replacement indicators. Moreover, in both cases, only few data elements were “one mouse click away” (poorly accessible), indicating a large amount of labor to extract all the required data from stand-alone computers and (paper) medical records. In case of automatic availability, manual collection was still necessary to complete the computation. Together with the overall under reporting of the required data elements, this leads to more implausible, estimated scores in the HKR case. That is, when HKR data was unavailable or difficult to access, hospitals did not withdraw from submitting indicator scores but estimated their indicator score to be 100%. In contrast, the CCC employees, who have access to items that are unavailable for the hospitals, (for instance for the data elements of “percentage of patients with recurrences within 5 years after surgery”) covered most of this labor intensive work for the BC PIs, and therefore, that data is less implausible. It has to be noted here, that many hospitals preferred to use their own indicator scores, when available, instead of that of the CCC even if the data was poorly accessible. As hospitals are free to choose which scores to be uploaded, we conclude that for both indicator sets the heterogeneous data collection and indicator computation largely affects the comparability of hospital performance.

### Policy implications

A governance model that increasingly relies on performance information as the basis for policy decisions (e.g. directly through selective contracting and indirectly through transparency of performance of care providers in the media), assumes the existence of high quality, reliable and valid performance information. Interestingly, this study has shown that the accuracy and precision of the PIs is questionable and further improvement of the current local hospital data-infrastructure in the Netherlands is necessary. There are several bottlenecks that need to be dealt with, ranging from the patchwork of hospital information systems, to the lack of a data-quality feedback loop back to the government.

Our results suggest that a nationally organized registry (in the case of breast cancer) led to more plausible indicator scores. Having one entity responsible for the data collection and indicator computation increases the comparability of the hospitals performance scores. Within the hip and knee replacement care, a fully operational and nationally coordinated medical registry does not yet exist. Therefore, hospitals are entirely dependent on their own local data infrastructure. As a result, many hospitals choose to upload indicator scores that are estimated on basis of locally implemented treatment protocols and not on basis of empirical observations, neither of the entire population, nor of a representative sample. In the latter case, one would expect lower indicator scores (score < 100%), as the timely administration of antibiotic prophylaxis does not often exceed 70% [13-15]. Estimating the indicator score instead of withdrawing from reporting altogether might be explained by the experienced external pressure caused by for example ranking lists that are published by the lay press. Hospitals end up at the bottom of the ranking when no indicator score is available, hence reporting 100% might be considered a favorable strategy to prevent reputation damage. Nevertheless, other factors such as a lack of priority or understaffing might additionally be at work as hospitals estimated their indicator score even though the data was available and reasonably accessible. The reporting of estimated data needs to be prevented as it results in a unrepresentative reflection for the quality of care delivered. Clinicians could for example set up a systematic peer review and consensus conference to discuss the PI scores before submitting them to the public database.

Despite the positive effect of the National Cancer Registry (NCR) on the Breast Cancer indicator plausibility, more profound standardization of these processes remains warranted. Particularly as governmental (DHTP) regulation regarding the data sources and the software systems that should be used for data collection and indicator computation is still lacking. This allows hospitals to choose their own strategies, which decreases the comparability of performance between hospitals. An alternative solution might be provided by disease specific registries that appear to be effective in improving health care quality and reducing costs, through publically available outcomes of health care [17]. Recently, several diagnoses specific medical registries have been set up in the Netherlands (e.g. Dutch Surgical Colorectal Audit [18]). Such a system avoids problems which arise when combining different data sources such as administrative data that can be easily calculated, with those based on other specific internal sources that can be easily manipulated. A drawback, however, is that a registry is set up from a unilateral, health care professional approach. The Netherlands has chosen to develop performance indicators according to a consensus driven perspective, implying that e.g. patient organizations and health care insurers are involved in the indicator selection and development process. Nevertheless this perspective might have led to a situation that methodological arguments for indicator selection and refinement lost too easily from political arguments to reach consensus on indicators that are supported by a broad selection of stakeholders. A consensus approach might benefit from more regulation with respect to the data quality, for instance by developing a data quality control framework that encompasses the most crucial steps of prevention, detection and actions to be taken with respect to insufficient data quality [19], particularly when using administrative data systems. It has been suggested that administrative data alone is not always appropriate for the valid computation of performance measures [10,20]. Moreover, a language formalization process of all the relevant items during the indicator development phase seems vital as in the Netherlands every hospital collects its own data, has its own local data infrastructure, and DHTP has no insight in the underlying data that hospitals submit [21,22]. After such a formalization process it could additionally be suggested to improve already existing national databases such as the Dutch Hospital Discharge registry or financial databases that are hosted by health care insurance companies.

Finally, the consensus approach entails that the indicators are used for several goals such as benchmarking performance, pay for performance schemes, selective contracting by insurance companies, and decision-making processes of patients looking for a healthcare provider. Particularly in the case of self-reported data, it should be made clear which indicators can be used for which specific goal.

## Conclusion

Our study provided insight in how performance indicator scores can be affected by heterogeneity of hospital information systems, data collection and data computation methods; factors that influence the reliability. Therefore, indicator developers, users and the scientific field need to focus more on the complexity of health care measurement instruments and conditional data infrastructures. Countries that have a liberal software market and are aiming to implement a self-report based performance indicator system to obtain health care transparency, should secure the accuracy and precision of the heath care data from which the PIs are calculated from. Moreover, ongoing research and development of PIs and profound insight in the clinical practice of data registration is warranted.

## Endnotes

^a^In Dutch: Zichtbare Zorg.

^b^According to the CCMO (Central Committee on Research involving Human Subjects), no medical-ethical approval of the study was necessary.

## Abbreviations

PI: Performance indicator; HKR: Hip and knee replacements; HR: Hip replacements; KR: Knee replacements; DHTP: Dutch health care transparancy program; CCC: Comprehensive cancer centers; CCMO: Central committee on research involving human subjects.

## Competing interests

The authors declare that they have no competing interests.

## Authors’ contributions

We confirm that the manuscript has been read and approved by all named authors and that there are no other persons who satisfied the criteria for authorship but are not listed. We further confirm that the order of authors listed in the manuscript has been approved by all of us. All authors contributed to the design of the study and assisted to draft and critically revise the manuscript. HA led the effort to set up and execute the research with the assistance of CF. HA and JK were substantially involved in preparing the manuscript. ES and NK additionally advised with their experience and expertise in performance indicator research.

## Pre-publication history

The pre-publication history for this paper can be accessed here:

http://www.biomedcentral.com/1472-6963/13/212/prepub

## Supplementary Material

Additional file 1 Overview of process and outcome indicators hip and knee replacements and breast cancer: numerators and denominators. Click here for file

## References

[B1] Mainz J Defining and classifying clinical indicators for quality improvement Int J Qual Health Care 2003 15 6 523 530 10.1093/intqhc/mzg081 14660535

[B2] Williams SC Watt A Schmaltz SP Koss RG Loeb JM Assessing the reliability of standardized performance indicators Int J Qual Health Care 2006 18 3 246 255 10.1093/intqhc/mzi098 16431865

[B3] Wollersheim H Hermens R Hulscher M Braspenning J Ouwens M Schouten J Marres H Dijkstra R Grol R Clinical indicators: development and applications Neth J Med 2007 65 1 15 22 17293635

[B4] van Dishoeck AM Lingsma HF Mackenbach JP Steyerberg EW Random variation and rankability of hospitals using outcome indicators BMJ Qual Saf 2011 20 10 869 874 10.1136/bmjqs.2010.048058 21642443

[B5] Egol A Shander A Kirkland L Wall MH Dorman T Dasta J Bagwell S Kaufman D Matthews P Jr Greenwald BM Herr DL Stavish C Thompson C Fahy BG Society of Critical Care Medicine Pay for performance in critical care: an executive summary of the position paper by the Society of Critical Care Medicine Crit Care Med 2009 37 9 2625 2631 10.1097/CCM.0b013e3181b4c3ad 19687632

[B6] Groene O Skau JK Frolich A An international review of projects on hospital performance assessment Int J Qual Health Care 2008 20 3 162 171 10.1093/intqhc/mzn008 18339665

[B7] Ogbu UC Westert GP Slobbe LC Stronks K Arah OA A multifaceted look at time of admission and its impact on case-fatality among a cohort of ischaemic stroke patients J Neurol Neurosurg Psychiatry 2011 82 1 8 13 10.1136/jnnp.2009.202176 20667853

[B8] Fischer C Anema HA Klazinga NS The validity of indicators for assessing quality of care: a review of the European literature on hospital readmission rate Eur J Public Health 2011 22 4 484 491 22140251 10.1093/eurpub/ckr165

[B9] Pronovost PJ Goeschel CA Viewing health care delivery as science: challenges, benefits, and policy implications Health Serv Res 2010 45 5 Pt 2 1508 1522 21054369 10.1111/j.1475-6773.2010.01144.x PMC2965889

[B10] Booth JL Collopy BT A national clinical indicator database: issues of reliability and validity Aust Health Rev 1997 20 4 84 95 10.1071/AH970084 10178134

[B11] Pringle M Wilson T Grol R Measuring “goodness” in individuals and healthcare systems BMJ 2002 325 7366 704 707 10.1136/bmj.325.7366.704 12351367 PMC1124227

[B12] Huff ED Comprehensive reliability assessment and comparison of quality indicators and their components J Clin Epidemiol 1997 50 12 1395 1404 10.1016/S0895-4356(97)00218-7 9449943

[B13] Lundine KM Nelson S Buckley R Putnis S Duffy PJ Adherence to perioperative antibiotic prophylaxis among orthopedic trauma patients Can J Surg 2010 53 6 367 372 21092428 PMC2993037

[B14] van Kasteren ME Kullberg BJ de Boer AS Mintjes-de GJ Gyssens IC Adherence to local hospital guidelines for surgical antimicrobial prophylaxis: a multicentre audit in Dutch hospitals J Antimicrob Chemother 2003 51 6 1389 1396 10.1093/jac/dkg264 12746377

[B15] Stefansdottir A Robertsson O Dahl A Kiernan S Gustafson P Lidgren L Inadequate timing of prophylactic antibiotics in orthopedic surgery. We can do better Acta Orthop 2009 80 6 633 638 10.3109/17453670903316868 19995312 PMC2823303

[B16] Dutch Orthopedic Society Diagnosis and treatment of hip and knee arthrosis 2007 [http://www.cbo.nl/Downloads/363/]

[B17] Larsson S Lawyer P Garellick G Lindahl B Lundström M Use of 13 disease registries in 5 countries demonstrates the potential to use outcome data to improve health care’s value Health Affairs 2012 31 220 227 22155485 10.1377/hlthaff.2011.0762

[B18] van GW Krijnen P Lemmens VE den DM Putter H van de Velde CJ Quality assurance in rectal cancer treatment in the Netherlands: a catch up compared to colon cancer treatment Eur J Surg Oncol 2010 36 4 340 344 10.1016/j.ejso.2009.10.010 19944552

[B19] Arts DG De Keizer NF Scheffer GJ Defining and improving data quality in medical registries: a literature review, case study, and generic framework J Am Med Inform Assoc 2002 9 6 600 611 10.1197/jamia.M1087 12386111 PMC349377

[B20] Iezzoni LI Using administrative data to study persons with disabilities Milbank Q 2002 80 2 347 379 10.1111/1468-0009.t01-1-00007 12101876 PMC2690114

[B21] Dentler K Cornet R Ten Teije A De Keizer NF Comparison of Reasoners for large Ontologies in the OWL 2 EL Profile SEMANTIC WEB 2012 2 2 71 87

[B22] Medlock S Opondo D Eslami S Askari M Wierenga P de Rooij SE Abu-Hanna A LERM (Logical Elements Rule Method): a method for assessing and formalizing clinical rules for decision support Int J Med Inform 2011 80 4 286 295 10.1016/j.ijmedinf.2011.01.014 21333589

