# Supplementary material for: Influences of hospital information systems, indicator data collection and computation on reported Dutch hospital performance indicator scores
Source: BMC Health Serv Res. 2013 Jun 12;13:212. doi: 10.1186/1472-6963-13-212 (PMC3698115; doi:10.1186/1472-6963-13-212)
Supplement: Additional file 1 — Overview of process and outcome indicators hip and knee replacements and breast cancer: numerators and denominators. [file 1472-6963-13-212-S1.docx]

**Additional file 1**

Table S1: National Performance Indicators: numerators and denominators

|  | | | | |
| --- | --- | --- | --- | --- |
|  | **Total Hip and Knee replacements *** | **S** | **P** | **O** |
| **2b** | % of patients that was administered thrombosis prophylaxis for 6 weeks to 3 months post-surgery, in case of total hip or knee surgery |  | X |  |
| **Num** | Number of patients that received thrombosis prophylaxis for 6 weeks to 3 months post- total hip or knee surgery |  |  |  |
| **Den** | Number of patients that underwent total knee or total hip replacement in a certain calendar year |  |  |  |
| **4b** | % of patients that **did not** (2008 & 2009)/ **did** (2010) receive a homologue blood transfusion, in case of total hip or knee surgery |  | X |  |
| **Num** | Number of patients that received (2008 & 2009)/ did not receive (2010) a homologue blood transfusion |  |  |  |
| **Den** | Number of patients that underwent total knee or total hip replacement in a certain calendar year |  |  |  |
| **5b** | % of patients that was administered antibiotics perioperatively |  | X |  |
| **Num** | Number of patients that was administered antibiotic prophylaxis, peri-operatively. |  |  |  |
| **Den** | Number of patients that underwent total knee or total hip replacement in a certain calendar year |  |  |  |
| **5c** | % of patients that was administered antibiotics 15 to 60 min. prior to surgery or to blood emptiness |  | X |  |
| **Num** | Number of patients that was administered antibiotics 15 to 60 min. prior to total hip or knee surgery or to blood emptiness |  |  |  |
| **Den** | Number of patients that underwent total knee or total hip replacement in a certain calendar year |  |  |  |
| **5d** | % of patients with a deep wound infection after a total hip or knee replacement |  |  | X |
| **Num** | Number of patients with a deep wound infection after a total hip or knee replacement |  |  |  |
| **Den** | Number of patients that underwent a total knee or total hip replacement in a certain calendar year |  |  |  |
|  | **Breast Cancer **** | **S** | **P** | **O** |
| **1** | % patients who were seen by a breast cancer nurse specialist preoperatively |  | X |  |
| **Num** | Number of patients with a breast tumor that had at least one preoperative meeting with a breast cancer nurse specialist |  |  |  |
| **Den** | Total number of patients with primary surgery of a breast tumor |  |  |  |
| **2** | % patients that was reviewed preoperatively in a multi-disciplinary team meeting |  | X |  |
| **Num** | Number of patients with a breast tumor that was reviewed in a documented, multi-disciplinary team meeting, prior to any treatment |  |  |  |
| **Den** | Total number of patients that was diagnosed with a breast tumor |  |  |  |
| **3** | % patients with a non-radical primary tumor resection |  |  | X |
| **Num** | Number of patients with a non-radical, primary tumor resection (breast saving surgery) |  |  |  |
| **Den** | Total number of patients with a primary tumor resection (breast saving surgery) |  |  |  |
| **4** | % surgeons in the surgery department that perform surgical treatments of breast tumors | X |  |  |
| **Num** | Number of surgeons in department that perform surgical treatments of breast tumors |  |  |  |
| **Den** | Total number of surgeons in department |  |  |  |
| **5** | % patients that are operated within 4 weeks after the final lab results are known |  | X |  |
| **Num** | Number of patients that are operated within 4 weeks after the final lab results are known |  |  |  |
| **Den** | Total number of patients with a primary tumor resection |  |  |  |
| **6a** | % patients with local recurrences within 5 years after breast-conserving surgery |  |  | X |
| **Num** | Number of patients with local recurrences within 5 years after breast-conserving surgery, primarily treated in own center (no referral) |  |  |  |
| **Den** | Total number of patients with breast conserving therapy, primarily treated in own center (no referral) |  |  |  |
| **6b** | % patients that have local recurrences within 5 years after ablative breast surgery |  |  | X |
| **Num** | Number of patients with local recurrences within 5 years after ablative breast surgery, primarily treated in own center (no referral) |  |  |  |
| **Den** | Total number of patients with ablative breast surgery, primarily treated in own center (no referral) |  |  |  |
| **7** | % of patients with a breast tumor that was postoperatively reviewed in a documented multi-disciplinary team meeting | X |  |  |
| **Num** | Number of patients that was postoperatively reviewed in a documented multi-disciplinary team meeting |  |  |  |
| **Den** | Total number of patients with breast surgery |  |  |  |

** Note: 5 yes/no “Hip/Knee structure indicators” are omitted from the table as they were not included in the current study; ** Indicators 1,2 and 7 were removed from the indicator set in 2009, 4 in 2011; S = structure, P = process, O = intermediate outcome; The PIs consist of numerators and denominators that each are composed of several variables according to combinatory logic that is described in instruction manuals.*
